# Genome-wide significant results identified for plasma apolipoprotein H levels in middle-aged and older adults

**DOI:** 10.1038/srep23675

**Published:** 2016-03-31

**Authors:** Karen A. Mather, Anbupalam Thalamuthu, Christopher Oldmeadow, Fei Song, Nicola J. Armstrong, Anne Poljak, Elizabeth G. Holliday, Mark McEvoy, John B. Kwok, Amelia A. Assareh, Simone Reppermund, Nicole A. Kochan, Teresa Lee, David Ames, Margaret J. Wright, Julian N. Trollor, Peter W. Schofield, Henry Brodaty, Rodney J. Scott, Peter R. Schofield, John R. Attia, Perminder S. Sachdev

**Affiliations:** 1 Centre for Healthy Brain Ageing, School of Psychiatry, University of New South Wales, Sydney, Australia; 2 Hunter Medical Research Institute, University of Newcastle, Newcastle, Australia; 3 Mathematics and Statistics, Murdoch University, Perth, Australia; 4 Bioanalytical Mass Spectrometry Facility, University of New South Wales, Sydney, Australia; 5 School of Medical Sciences, University of New South Wales, Sydney, Australia; 6 Centre for Clinical Epidemiology & Biostatistics, University of Newcastle, Newcastle, Australia; 7 Neuroscience Research Australia, Randwick, Australia; 8 School of Medical Sciences, University of New South Wales, Sydney, Australia; 9 Department of Developmental Disability Neuropsychiatry, University of New South Wales, Sydney, Australia; 10 Neuropsychiatric Institute, Prince of Wales Hospital, Randwick, Australia; 11 National Ageing Research Institute, Melbourne, Australia; 12 Academic Unit for Psychiatry of Old Age, University of Melbourne, Melbourne, Australia; 13 Queensland Brain Institute, University of Queensland, Brisbane, Australia; 14 School of Medicine & Public Health, University of Newcastle, Newcastle, Australia; 15 Dementia Collaborative Research Centre – Assessment and Better Care, University of New South Wales, Sydney, Australia

## Abstract

Apolipoprotein H (ApoH) is a multi-functional plasma glycoprotein that has been associated with negative health outcomes. ApoH levels have high heritability. We undertook a genome-wide association study of ApoH levels using the largest sample to date and replicated the results in an independent cohort (total N = 1,255). In the discovery phase, a meta-analysis of two cohorts, the Sydney Memory and Ageing Study (Sydney MAS) and the Older Australian Twins Study (OATS) (n = 942) revealed genome-wide significant results in or near the *APOH* gene on chromosome 17 (top SNP, rs7211380, p = 1 × 10^−11^). The results were replicated in an independent cohort, the Hunter Community Study (p < 0.002) (n = 313). Conditional and joint analysis (COJO) confirmed the association of the chromosomal 17 region with ApoH levels. The set of independent SNPs identified by COJO explained 23% of the variance. The relationships between the top SNPs and cardiovascular/lipid/cognition measures and diabetes were assessed in Sydney MAS, with suggestive results observed for diabetes and cognitive performance. However, replication of these results in the smaller OATS cohort was not found. This work provides impetus for future research to better understand the contribution of genetics to ApoH levels and its possible impacts on health.

Apolipoproteins are a family of multifunctional glycoproteins that bind and transport lipids in the circulatory system. Apolipoprotein H (ApoH), also known as β2-glycoprotein I (β2GPI), is a 43–50 kDa single-chain glycoprotein that is expressed predominantly in the liver but also in other cell types such as endothelial cells, lymphocytes, astrocytes and neurons^1^,^2^. ApoH is a phospholipid-binding (e.g. cardiolipin) plasma protein that also binds other negatively charged molecules such as DNA and oxidised low-density lipoproteins and interacts with a variety of proteins^3^,^4^. It is involved in a number of physiological processes including lipid metabolism, coagulation, apoptosis, host defence against bacterial infections, inflammation, angiogenesis and atherogenesis^3^,^5^,^6^,^7^. Importantly, ApoH is the main target of anti-phospholipid antibodies (aPLs) found in many patients with systemic lupus erythematosus and antiphospholipid syndrome (APS)^3^,^7^,^8^,^9^. APS is characterised by thrombosis and pregnancy complications including miscarriage. aPLs, which include those directed against ApoH, are also found in a small proportion of the general population and become more common with ageing^10^,^11^,^12^. ApoH levels have also been linked to cognitive ageing, the predementia syndrome, mild cognitive impairment, and Alzheimer’s disease^13^,^14^,^15^,^16^,^17^. Thus ApoH is potentially involved in a wide range of health outcomes and a better understanding of the factors influencing its levels is required to elucidate the roles ApoH may play in disease.

Genetics plays a strong role in determining ApoH levels as demonstrated by the high heritability (78%) of plasma ApoH levels^18^. The *APOH* gene is located on chromosome 17 and has 8 exons^19^,^20^. Previous studies have demonstrated that variation in the *APOH* gene influences plasma ApoH levels. Two missense *APOH* single nucleotide polymorphisms (SNPs) (rs1801692 [Ser107Asn, also known as Ser88Asn], rs1801690 [Trp335Ser, also known as Trp316Ser]) explained up to 14% of the observed plasma ApoH variation in Caucasians with a mean age of ~53 years^7^,^21^. Rs1801690 and another missense SNP, rs1801689 [Cys325Gly, also known as Cys306Gly], were also associated with ApoH levels in lupus patients^22^. Furthermore, rs1801690 and three other *APOH* SNPs in high linkage disequilibrium (LD) were associated with increased risk of venous thrombosis and ApoH levels in a Chinese sample^23^. In a later study, Mehdi *et al*.^24^ observed reduced gene expression and lower plasma ApoH levels for the minor allele of an *APOH* polymorphism located next to the transcriptional initiation site (-1C- > A), rs8178822, which may interfere with the binding of the transcription factor, TFIID^24^. In addition, Suresh *et al*.^25^ have described several *APOH* promoter SNPs that influence transcription *in vitro*, including rs8178822, rs3760292 (−643 from translation start) and rs8178819 (−1219 from translation start). However, when the relationships between these SNPs were investigated with ApoH levels in human blood; only rs8178822 was significant, which may reflect the differences between *in vitro* experiments and the more complex *in vivo* human samples.

One previous genome-wide association study (GWAS) has been reported but used a small family sample (N = 306) and did not have a healthy replication sample^7^. The authors examined the associations of plasma ApoH levels with over 300,000 SNPs. Three SNPs reached genome-wide significance (p ≤ 1.76 × 10^−7^), which were located in the *ELF5* and *SCUBE2* genes. Suggestive evidence was observed for several SNPs, including a variant located in the *APOH* gene and several SNPs located nearby. The three genome-wide significant results were then investigated further by examining their association with coronary artery disease (CAD) using a large sample (n = 5,765 CAD; n = 7,624 controls) with two linked SNPs, located in the *ELF5* gene region, nominally significant.

In this study, we aimed to identify novel genetic variants associated with ApoH levels by undertaking a larger GWAS in a sample of middle-aged to older adults. The discovery phase was undertaken in two Australian cohorts of older adults (n = 942). Genome-wide significant results for ApoH levels were observed in or near the *APOH* gene on chromosome 17. These results were replicated in an independent study of middle-aged to older adults (n = 313). Furthermore, the relationship between the identified SNPs and cardiovascular-related phenotypes, diabetes and cognitive performance was also assessed.

## Results

Descriptive statistics for the three cohorts are shown in Table 1. Of the three cohorts, the Sydney Memory and Ageing Study (Sydney MAS) cohort was the oldest and the Hunter Community Study (HCS) the youngest, as it included individuals aged 55 years and over. Current use of hypolipidemic medication was common in the two older cohorts (Sydney MAS, Older Australian Twin Study [OATS]).

### Genome-wide Association Analyses (GWAS)

**Figure 1:**
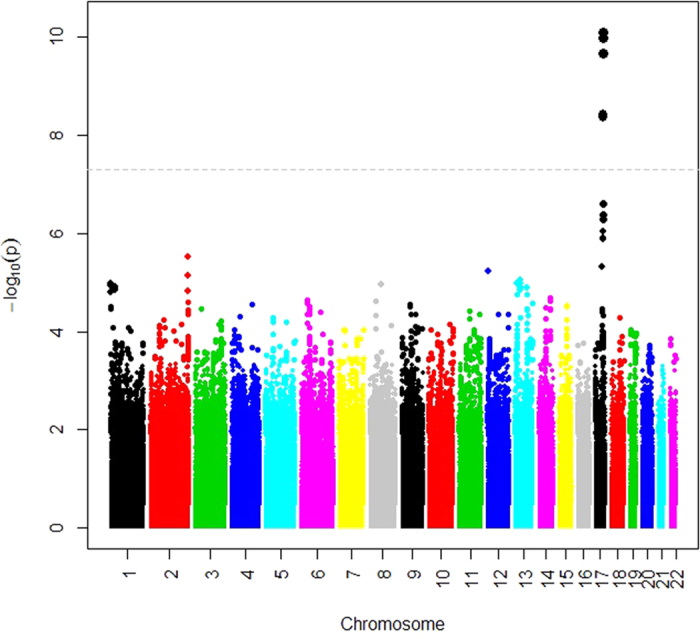
Manhattan Plot: Results of the Discovery Plasma ApoH GWAS (Model 2 = age, sex, assay batch, hypolipidemic medication). P-values for each individual SNP are plotted against chromosome position from the results of the discovery association analyses for plasma ApoH levels.

**Figure 2:**
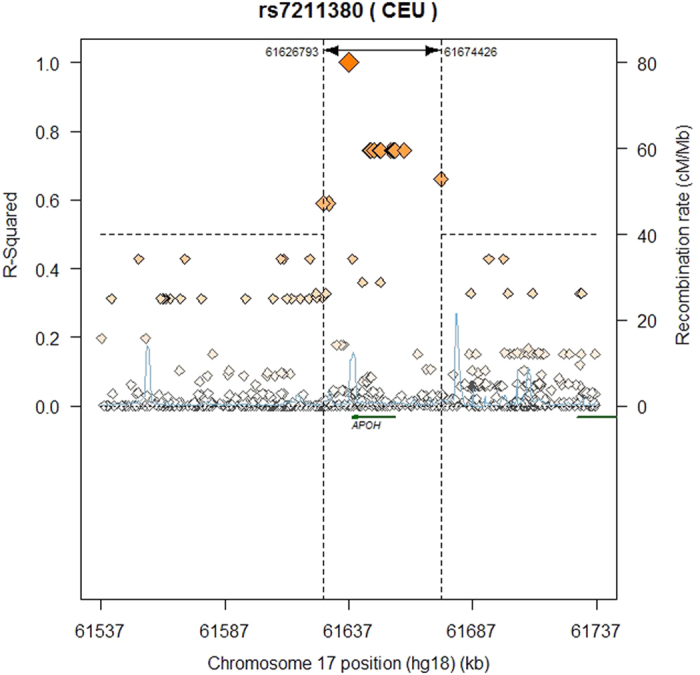
Linkage disequilibrium plot for the top ApoH genome-wide significant SNP (rs7211380) and surrounding SNPs in a 200 base pair window based on the 1K Genomes reference panel using CEU samples.

GWAS were undertaken using linear regression models in each of the cohorts. In the discovery phase using a meta-analysis of the GWAS results from the two cohorts, Sydney MAS and OATS, twelve genome-wide significant SNPs were observed (p < 5 × 10^−8^) when age, sex and hypolipidemic medication were used as covariates (Model 2, Fig. 1, Table 2). The results for Model 1 (only age, sex and batch number as covariates) are shown in Supplementary Tables S1 and S2, with most of the results overlapping between the two models. All of the genome-wide SNPs were located in or close to the *APOH* gene region and in general were in high linkage disequilibrium (Table 2, Fig. 2). Replication of the genome-wide significant results was undertaken in HCS and all SNPs showed evidence of replication (p < 0.002, Table 2), even after Bonferroni correction (p = 0.05/12 tests = 0.004).

The most significant SNP (rs7211380) was located 3′ to the *APOH* gene and explained 3.7% of the variance (see Table 2). The other *APOH* SNPs were spread across the gene including a non-synonymous SNP, which results in the substitution of arginine with histidine at amino acid 154 (rs817884, also known as Arg135His). As shown in Table 3, several suggestive SNPs in the *CEP112* gene (p < 1 × 10^−5^) also showed evidence of replication in the HCS (p ≤ 0.02), but did not survive Bonferroni correction for multiple testing (p = 0.05/13 tests = 0.004). The GWAS discovery summary results are available at https://www.cheba.unsw.edu.au/group/genetics-genomics.

### Conditional and Joint Analyses

To investigate further a conditional and joint analysis (COJO) was undertaken using the meta-analysis results for Sydney MAS and OATS, when adjusted for age, sex, assay batch number and hypolipidemic medication. In the final model (see Supplementary Table S3), only chromosomal 17 SNPs were identified, including SNPs not identified in the meta-analysis (cf Tables 2 and 3). These SNPs were spread over a 10,227,682 bp region containing the *APOH, CEP112* and *PRKCA* genes and explained 23% of the variance in ApoH levels.

### Replication of prior genetic results

#### Prior Genetic Studies

Six SNPs located in or near the *APOH* gene have been previously associated with ApoH levels using *in vitro* experiments and/or human samples. These SNPs are rs1801692 (exon 3, Ser107Asn), rs1801690 (exon 8, Trp335Ser)^21^, rs3760292 (−632 from transcription start site), rs8178819 (−1219 from transcription start site), the missense SNP rs4791077 now known as rs1801689 (Cys325Gly) and rs8178822 (5′ UTR, −32 from translational start site)^24^.

Rs8178822, was identified as a genome-wide significant hit in our GWAS. Rs3760292 was available in all three cohorts but was not significantly associated with ApoH levels (p > 0.311). The other four SNPs (rs1801690, rs1801692, rs8178819, rs1801689) were only available in OATS and HCS. Rs1801690, was significant in both OATS (p = 5.53 × 10^−6^) and HCS (p = 0.0001) as was rs1801689 (OATS, p = 0.002; HCS p = 2.46 × 10^−5^) and the results were in the same direction in both cohorts. The SNP rs8178819 approached nominal significance (OATS p = 0.075, HCS p = 0.053) but rs1801692 was not significant (p > 0.17).

#### Prior GWAS

Of the ten SNPs identified in the previously published GWAS (see Supplementary Table S4)^7^, only three SNPs showed suggestive evidence of replication in the Sydney MAS/OATS meta-analysis (rs2647528 p = 0.008, rs7209395 p = 0.037, rs10048158 [*APOH*], p = 0.016) but none survived Bonferroni correction for multiple testing.

### Investigation of top SNPs with cardiovascular phenotypes, diabetes and cognition

The associations of the top 25 SNPs identified in the GWAS (genome-wide significant plus suggestive) with cardiovascular phenotypes (myocardial infarction, stroke, hypertension, HDL and LDL cholesterol levels), diabetes and cognitive performance were examined. In Sydney MAS, two SNPs were nominally associated with type 2 diabetes (rs2010251, rs758767), with an odds ratio of 1.67 (p = 0.015) (see Table 4). The SNP rs2873966 was associated with the cognitive domains of attention/processing speed (p = 0.001), executive function (p = 0.014) and global cognition (p = 0.001). Two other SNPs also showed evidence of association with attention/processing speed (rs2010251, rs758767). Replication of these results was undertaken in OATS with one SNP approaching nominal significance (rs287396) for the cognitive domain of attention/processing speed (p = 0.054) and showing nominal significance for global cognition (p = 0.047). However, these results were in the opposite direction and were not significant after adjusting for multiple testing.

## Discussion

In our study we investigated the genetics of plasma apolipoprotein H levels in community-dwelling cohorts of older adults using a GWAS meta-analysis. Our results were replicated in an independent cohort of middle-aged to older adults. Consistent with prior results we observed SNPs in and around the *APOH* gene on chromosome 17 that contributed to the variance observed in plasma ApoH levels. Furthermore, when we undertook a conditional and joint analysis, only a region on chromosome 17 was significantly associated with ApoH levels. The joint analysis identified new SNPs that were not GWAS-significant in the single SNP analysis. These SNPs explained 23% of the variance in ApoH levels. We extended these analyses to examine whether these SNPs were associated with diabetes, cardiovascular and cognitive phenotypes. Indeed, several *APOH* SNPs were nominally associated with diabetes and cognitive performance in the Sydney MAS but not in the smaller OATS cohort. We also replicated prior candidate gene results for the missense variants, rs1801690 and rs1801689.

Most of the identified *APOH* GWAS SNPs are in linkage disequilibrium. Interestingly, our top SNP, rs7211380, was not in linkage disequilibrium with the suggestive *APOH* SNP observed by Athaniasadis *et al*.^7^ (rs10048158, r^2^ = 0.046). This SNP (rs7211380) is located 3′ to the *APOH* gene. However, previous work provides clues to the SNPs that may be driving the observed relationships in this study. For example, the minor allele of the 5′ UTR genome-wide significant SNP, rs8178822, located next to the transcription start site, has been previously linked to lower gene and protein expression *in vitro* and *in vivo*^24^,^25^. Moreover, our results were in a similar direction to that previously observed^24^. Our top SNP rs7211380 is in high LD with rs8178822 (r^2^ = 0.90). Another top GWAS SNP of interest is the non-synonymous coding SNP, rs8178847, which results in the substitution of arginine with histidine, which may have implications for the integrity of the protein. However, analysis using SIFT^26^, suggested this amino acid substitution would not be deleterious to protein function. This SNP has previously been associated with body mass index in type 2 diabetic patients receiving thiazolidinedione therapy^27^. Using a gene-based analysis VEGAS^28^, only the *APOH* locus was significant (p = 1 × 10^−7^, data not shown). In addition, pathway analysis of our top GWAS results using software packages such as MAGENTA^29^ and iGSEA4GWAS^30^ did not reveal any significantly enriched pathways (results not shown) as most of our top results were observed in and around the *APOH* gene on chromosome 17.

By undertaking a conditional and joint analysis we were able to identify independent SNPs on chromosome 17 that were not genome-wide significant in the single SNP meta-analysis. For example, the *CEP112* intronic SNP rs9897921 had a p-value of 0.00012 in the single SNP analysis but in the joint analysis had a p-value of 2.8 × 10^−220^. This analysis implicated a wide region of chromosome 17 (10,226 kb). However, the majority of SNPs were located in a region of 617 kb encompassing not only *APOH* but also the *CEP112* and *PRKCA* genes. The remaining two SNPs were located in an intergenic region and were not in LD with our top SNP, rs7211380 (r^2^ = 0). Overall, the identified SNPs in this analysis explained a relatively large proportion of the phenotypic variance (23%).

None of the ten SNPs identified from the previous GWAS^7^ were significant in our discovery meta-analysis. However, three SNPs showed suggestive evidence of replication in our larger sample, one of which was located close to the *APOH* gene (rs10048158). Methodological differences between the two studies may contribute to the lack of replication of the prior GWAS results including the larger sample size of the present study (N = 942 discovery versus N = 306) and the different immunoassay methods used for measurement of ApoH levels. Our sample was older compared to the Athanasiadis study (M age 75.47 versus M age = 37.7 yrs) and the two studies assessed a different number of SNPs (greater in the current study). Although both studies used Caucasian samples and analysed the data similarly (additive genetic model, age and sex as covariates), we undertook an additional analysis including an extra covariate, hypolipidemic medication usage, which may influence ApoH levels that was not considered in the Athanasiadis GWAS. In contrast, we were able to replicate the prior association of the missense SNP, Trp335Ser (rs1801690), with the direction of the results consistent to that previously reported^24^. In addition, we also replicated a finding for the rare missense SNP, rs1801689 (Cys325Gly), which has previously been linked to ApoH levels in lupus patients^22^, to venous thrombosis and ApoH levels in a Chinese sample^23^ and to LDL cholesterol levels^31^. Interestingly, the amino acid changes from these two SNPs alter the binding capacity of ApoH for phospholipids^32^.

We found several of the top *APOH* SNPs located on chromosome 17 were also nominally associated with diabetes type 2 and cognitive performance in older adults in the full Sydney MAS cohort. Replication of these results in the smaller OATS cohort found only nominally significant results for the SNP rs2873966 and cognitive performance (attention/processing speed) that did not survive multiple testing correction. Moreover, the effects in OATS were not in the same direction. However, based on the Sydney MAS results, power calculations suggest a sample size of 760 would be required for 80% power. The OATS cohort used for these analyses was considerably smaller and hence larger samples are required to replicate these results.

A strength of this study is the use of the same immunoassay for ApoH across studies and that all assays were undertaken in the same laboratory, thus minimising phenotypic variability between studies. On the other hand, a limitation of this study is the relatively small sample used for discovery and replication. However, given the prior genetic association studies using even smaller samples that have found significant relationships between SNPs and ApoH levels it is not surprising that we observed significant results in our larger population. Another limitation is that several of the previously identified candidate SNPs were not available in the MAS cohort (due to poor imputation quality) and hence their relationships with ApoH levels, cardiovascular and cognitive phenotypes were not investigated. We did not observe any other significant SNPs associated with ApoH levels apart from the *APOH* gene locus, suggesting that larger cohorts will be required to find other genes that may influence ApoH, which may be of smaller effect.

In conclusion, we found SNPs on chromosome 17, including the *APOH* gene region, that were associated with ApoH levels in two cohorts of older adults that replicated in a sample of middle aged to older adults. We also found suggestive evidence for associations between *APOH* SNPs and (i) diabetes type 2 and (ii) cognitive performance. This work lays the foundation to improve our understanding of the genetic regulation of ApoH levels and how such variation may contribute to chronic inflammatory disease, diabetes type 2, and age-related cognitive performance.

## Methods

### Samples

Three Australian cohorts of middle-aged to older adults were analysed (n = 1,255). All participants provided written informed consent and ethics approval was given by the appropriate committees. The methods performed in this project were carried out in accordance with the relevant guidelines and regulations. All studies collected a diverse set of data including demographic, neuropsychological, health and medical data and peripheral blood samples for protein and DNA analyses. Information on current use of hypolipidemic medication was also collected.

#### Discovery Cohorts (n = 942)

Participants were from the Sydney Memory and Ageing Study (Sydney MAS) and the Older Australian Twins Study (OATS).

##### Sydney MAS

Briefly, older adults aged 70–90 years were randomly recruited from the compulsory electoral rolls of two Sydney electorates. Exclusion criteria included a diagnosis of dementia, psychotic symptoms or diagnosis of schizophrenia/bipolar disease and progressive malignancy. The University of New South Wales and the Illawarra Area Health Service Human Research Ethics Committees approved the study. At baseline, there were 1,037 participants with a mean age of 78.84 years and 44.8% were men. A subsample with both plasma ApoH levels and genome-wide genotyping data were used in this study (n = 603). Full details of the study are provided elsewhere^33^.

##### OATS

Participants aged 65 years and over were recruited through the Australian Twin Registry and a recruitment drive. Exclusion criteria included a current psychosis diagnosis and insufficient English to complete the assessment. The Australian Twin Registry, University of New South Wales, University of Melbourne, Queensland Institute of Medical Research and the South Eastern Sydney and Illawarra Area Health Service all gave approval for the study. Following recruitment, there were 623 participants with a mean age of 70.77 years and 34.8% were men. A subsample with both ApoH and genotyping data was used in the current study (n = 339). For further details see Sachdev *et al*.^34^.

#### Replication cohort

Over 7,500 people were randomly selected from the compulsory electoral roll of Newcastle, Australia, and invited to participate in the Hunter Community Study (HCS). Exclusion criteria included an inability to speak English and living in a residential care facility. After recruitment, there were 3,207 participants with a mean age of 66.3 years and 49.6% were male. Ethics approval for the study was obtained from the Hunter New England Local Health District and University of Newcastle Human Research Ethics Committees. A subsample was used in the present study who had available both genome-wide genotyping and plasma ApoH data (n = 313). For more details see McEvoy *et al*.^35^.

### Apolipoprotein H (ApoH) Levels

Plasma ApoH levels were measured in the same laboratory using the same measurement method. ApoH levels were assayed using a multiplex bead fluorescence immunoassay (WideScreen Human CVD Panel1; Novagen, EMD Chemicals Inc, WI) as per the manufacturer’s instructions. Five microliters of plasma was diluted in 1:2,500 using the kit’s dilution buffer. Fluorescence was measured using a Bioplex system (Luminex 100, BioRad, Hercules, CA). More details are provided in Song *et al*.^17^.

### Cardiovascular Phenotypes and Diabetes

Diabetes, myocardial infarction, stroke, hypertension, cholesterol, HDL and LDL cholesterol levels were assessed. Diabetes was defined as self-report of a diagnosis by a doctor, current usage of diabetes medication or a fasting blood glucose ≥7.0mM. Participants reported whether they ever had been diagnosed by a doctor with the following: (i) a myocardial infarct; and/or (ii) stroke. Current hypertension was defined as previous diagnosis and current treatment for hypertension, systolic blood pressure ≥160 mmHg, or diastolic blood pressure ≥95 mmHg. HDL cholesterol levels were estimated in overnight fasting peripheral venous blood samples using standard enzymatic colorimetric methods on a COBAS 6000 Analyser (Roche, Indianapolis, USA). LDL cholesterol levels were calculated using the Friedewald formula. Current use of hypolipidemic medication was assessed from self-reported medication usage.

### Cognitive Phenotypes

An extensive battery of cognitive tests was administered to participants of both the Sydney MAS and OATS. Details of the specific tests used are given in Sachdev *et al*.^33^,^34^. Cognitive domain scores were calculated as an average of the z-scores of the relevant tests: attention/processing speed, memory, verbal memory, language, visuo-spatial ability and executive function. Global cognition scores were calculated as the average of all domain scores except for verbal memory.

### Genotyping

For the majority of participants DNA was extracted using standard procedures from peripheral blood samples. For the Sydney MAS and OATS cohorts where blood samples were not available, DNA was extracted from saliva samples or cell lines (<5%).

#### Sydney MAS

Genome-wide genotyping was undertaken using the Affymetrix Genome-wide Human SNP Array 6.0 (California, USA) at the Ramaciotti Centre, UNSW Australia. Of the 972 DNA samples available, six samples were not successfully genotyped (insufficient DNA/failed genotyping). Genotypes were called using the CRLMM package (v1.10.0) in R (v2.12.1) and release 31 SNP6 annotations were used. Genotyped SNPs were excluded if the call rate was <95%, if the p-value for Hardy-Weinberg equilibrium was <10^−6^, and/or if the minor allele frequency was <0.01%. One participant was excluded due to a poor genotyping call rate (<95%). Relatedness checks were undertaken using identity-by-descent analysis in PLINK^36^, three pairs of siblings were identified and only one of each pair was kept in the dataset. Sex checks were performed using PLINK, which confirmed recorded gender. Assessment of population stratification and identification of ethnic outliers was undertaken using EIGENSTRAT (Price *et al*., 2006). Ethnic outliers were excluded (n = 37) and 17 significant principal components were observed. After quality control checks, there were 925 Sydney MAS participants (45.1% male) with data for 734,550 SNPs and an average genotyping call rate of 99.5%. Imputation was undertaken using the HapMap2 reference data (release 22, build 36) and MaCH^37^,^38^. Poorly imputed SNPs (*R*^*2*^ ≤ 0.6) were excluded from further analyses.

#### OATS

Samples (n = 548) were genotyped at the University of Queensland Diamantina Institute, Australia, using the Illumina Omni Express array (California, USA) following the manufacturer’s instructions. Genotypes (n = 733,202) were called using Illumina Genome Studio V2011.1. Quality control procedures were the same as for Sydney MAS. No participants were excluded due to poor genotyping call rates. Using PLINK, relatedness (identity-by-descent) and sex checks were undertaken to confirm family relationships and sex. Six samples were excluded due to unexpected relatedness or incorrect sex. Using EIGENSTRAT 25 ethnic outliers were identified, which were omitted from further analysis and three significant principal components were observed. After quality control checks, there was genotyping data available on 646,791 SNPs for 517 participants (35.2% male) with a mean genotyping call rate of 99.9%. Imputation was undertaken using the HapMap2 reference data as described for Sydney MAS.

#### HCS

Genome-wide genotyping was undertaken using the Affymetrix Axiom Kaiser array following the recommended protocol. Quality control filters used were similar to Sydney MAS. SNPs were excluded if the genotyping call rate was <95%, the Hardy-Weinberg equilibrium p-value was <1 × 10^−6^, or the MAF <0.01%. Participants were excluded if the genotyping call rate was low (<95%) and if the sex checks were discordant with the recorded gender. Relatedness checks were undertaken and if first or second-degree relatives were identified, only one family member was retained in the analysis. After EIGENSTRAT analysis ethnic outliers were identified and omitted from further analysis, 17 significant principal components were observed. After completion of quality control checks, there were 2,088 participants (49.6% male) with data for 739,276 SNPs and a mean genotyping call rate of 99.1%. Imputation was undertaken using the HapMap2 reference data (release 22, build 36) and MaCH v1.0.16^37^,^38^.

### Statistical Analyses

#### Genome-wide Association Study (GWAS)

ApoH was square root transformed to improve normality. Using an additive genetic model and multiple linear regression, a GWAS was undertaken adjusting for age, sex and ApoH assay batch number (model 1). Model 2 was also adjusted for the use of hypolipidemic medication, a possible confounder. In MAS and OATS, the GWAS was undertaken using mach2qtl^37^,^38^ and merlin^39^, respectively. An inverse-variance weighted meta-analysis of the GWAS results was undertaken in Sydney MAS and OATS using METAL^40^. Principal components were not used to account for population stratification in the analysis as we had removed any ethnic outliers in both MAS and OATS and the meta-analysis genomic inflation factor was acceptable (0.995). Linkage disequilibrium between SNPs (r^2^) was calculated using PLINK.

Replication of genome-wide significant (p < 5 × 10^−8^) and suggestive results (p < 1 × 10^−5^) was performed in the HCS (n = 313) using linear regression, an additive genetic model and GenABEL^41^. Models 1 and 2 were adjusted for population substructure using the first 4 principal components.

#### Conditional and Joint Analysis

Conditional and joint analysis (COJO) as implemented in the program GCTA^42^ was used to select a subset of associated SNPs with p-values < threshold (p_0_ = 5 × 10^−8^). This method uses a reference panel of SNPs to estimate the LD between the SNPs and the meta-analysis summary statistics. The method starts with the top SNP (least p < p_0_) in the meta-analysis and then the p-values for all the remaining SNPs are calculated conditional on the selected SNP (top SNP). It then selects the next top SNP in the conditional analysis (p < p_0_) and proceeds to fit all the selected SNPs in the model dropping all those SNPs with p-values > p_0_. The iteration continues until no SNP is added or dropped from the model thus finding a subset of associated SNPs with a threshold for LD (r^2^ < 0.9) between SNPs. Finally, a joint analysis of the subset of associated SNPs is performed. For the COJO analysis we used the results from the ApoH meta-analysis (discovery results) and the MAS imputed data as the reference panel.

#### Replication of prior genetic results

Replication of prior results (N = 15 SNPs) from candidate gene studies and the previous GWAS was undertaken using an additive genetics model and linear regression. P-values < 0.003 were considered significant after Bonferroni correction for the number of statistical tests undertaken (p = 0.05/15 = 0.003).

### Analysis of Top *APOH* SNPs with cardiovascular-related and cognitive phenotypes

Analyses were undertaken in Sydney MAS using an additive model and linear (continuous variables) or logistic regression (categorical variables). Cardiovascular phenotypes and diabetes analyses examining the top 25 GWAS identified SNPs were undertaken using age and sex as covariates. For cognitive phenotypes, the additional covariates used were years of education and non-English speaking background. Replication of the top results (<0.05) was undertaken in the OATS cohort.

## Additional Information

**How to cite this article**: Mather, K. A. *et al*. Genome-wide significant results identified for plasma apolipoprotein H levels in middle-aged and older adults. *Sci. Rep.* **6**, 23675; doi: 10.1038/srep23675 (2016).

## Supplementary Material

Supplementary Information

## Figures and Tables

**Table 1:** Descriptive statistics for the discovery cohorts (Sydney Memory and Ageing Study, Older Australian Twins Study) and the replication cohort (Hunter Community Study).

	Sydney MAS	OATS	HCS
Sample size	603	339	313
Age (yrs), *M* (*SD*)	78.22 (4.54)	70.58 (5.24)	66.28 (8.06)
Number females (%)	337 (55.9)	202 (59.6)	144 (46.0)
Plasma ApoH Levels (ug/ml), median (IQR)	164.9 (IQR = 135.6–190.0)	136.87 (IQR = 94.76–170.0)	146.73 (IQR = 110.1–180.3)
Current hypolipidemic medication use N, (%)	288 (47.8)	123 (36.3)	97 (31.0)
Genotyping platform	Affymetrix 6.0	Illumina OmniExpress	Affymetrix Axiom Kaiser array

Sydney MAS = Sydney Memory and Ageing Study, OATS = Older Australian Twins Study, HCS = Hunter Community Study.

**Table 2:** Genome-wide significant plasma ApoH GWAS results for the discovery meta-analysis and the replication sample for Model 2 (age, sex, assay batch, hypolipidemic medication).

SNP (rs)	Chr	BP	Effect Allele (minor)	Beta (S.E.)	*Discovery* p-value	Direction	Variance Explained	Replication p-value (HCS)	Direction (HCS)	Gene	Feature
rs7211380	17	64206768	G	−1.20 (0.19)	8.15E-11	− −	0.037	0.000517	−	*APOH*	3′
rs11651658	17	64198640	C	−1.19 (0.18)	1.02E-10	− −	0.036	0.000872	−	NA	Near *APOH*
rs1014399	17	64196331	A	−1.16 (0.18)	2.20E-10	− −	0.035	0.001453	−	NA	Near *APOH*
rs8178853	17	64215058	A	−1.12 (0.19)	3.82E-09	− −	0.030	0.001138	−	*APOH*	intron
rs8178851	17	64215239	C	−1.12 (0.19)	3.85E-09	− −	0.030	0.001141	−	*APOH*	intron
rs8178841	17	64219197	A	−1.12 (0.19)	3.86E-09	− −	0.030	0.001157	−	*APOH*	intron
rs8178838	17	64219541	C	−1.12 (0.19)	3.92E-09	− −	0.030	0.001139	−	*APOH*	intron
rs16958979	17	64223859	T	−1.12 (0.19)	4.01E-09	− −	0.030	0.001116	−	*APOH*	intron
rs7213041	17	64224616	T	−1.12 (0.19)	4.01E-09	− −	0.030	0.001112	−	*APOH*	intron
rs8178847	17	64216815	T	−1.12 (0.19)	4.26E-09	− −	0.030	0.001157	−	*APOH*	missense (Arg –> His)
rs8178822	17	64225529	T	−1.12 (0.19)	4.27E-09	− −	0.030	0.001122	−	*APOH*	UTR 5′
rs8178842	17	64218640	T	−1.12 (0.19)	4.29E-09	− −	0.030	0.001157	−	*APOH*	Intron

Note: SNP annotation information from SNPnexus^43^; Genomic inflation factor (λ_GC_) for the discovery sample was 0.995; Variance explained = 2*p*q*beta^2^/Variance of ApoH, allele frequencies were estimated using Sydney MAS and ApoH variance from the discovery analysis; APOH = apolipoprotein H.

**Table 3:** Suggestive plasma ApoH GWAS results for the discovery meta-analysis and the replication sample for Model 2 (age, sex, assay batch effects, hypolipidemic medication).

SNP (rs)	Chr	BP	Effect Allele	Beta (S.E.)	*Discovery* p-value	Direction	Variance explained	Replication p-value (HCS)	Direction (HCS)	Gene	Feature
rs2873966	17	64211973	A	0.53 (0.10)	2.54E-07	+ +	0.025	0.068927	+	*APOH*	intronic
rs11655503	17	64027346	A	−0.97 (0.19)	4.24E-07	− −	0.028	0.010205	−	*CEP112*	intronic
rs8064837	17	64242703	G	−0.46 (0.09)	5.11E-07	− −	0.023	0.113782	−	N/A	N/A
rs1420791	17	63914750	G	−0.92 (0.19)	8.90E-07	− −	0.022	0.011073	−	*CEP112*	intronic
rs2010251	17	64203725	T	−0.61 (0.13)	1.26E-06	− −	0.022	0.03413	−	N/A	N/A
rs758767	17	64204591	A	−0.61 (0.13)	1.26E-06	− −	0.022	0.034137	−	N/A	N/A
rs7214750	17	63903119	T	−0.90 (0.19)	1.30E-06	− −	0.021	0.015964	−	*CEP112*	intronic
rs6431248	2	235202974	A	0.46 (0.10)	3.00E-06	+ +	0.021	0.722578	+	N/A	N/A
rs181247	17	56207731	A	0.58 (0.13)	4.88E-06	+ +	0.028	0.021183	+	N/A	N/A
rs12825437	12	5756980	A	−0.65 (0.14)	5.91E-06	− −	0.019	0.486807	+	*TMEM16B*	intronic
rs1519187	2	235201218	T	0.45 (0.10)	7.18E-06	+ +	0.02	0.876655	+	N/A	N/A
rs9562709	13	47727650	A	0.48 (0.11)	8.83E-06	+ +	0.018	0.488741	−	N/A	N/A
rs9591011	13	47728087	T	0.48 (0.11)	9.04E-06	+ +	0.017	0.488816	−	N/A	N/A

Note: SNP annotation information from SNPnexus^43^; Variance explained = 2*p*q*beta^2^/Variance of ApoH, allele frequencies were estimated using Sydney MAS and ApoH variance from the discovery analysis; *APOH*, apolipoprotein H; *CEP112,* centrosomal protein 112kD; *TMEM16B* (ANO2), anoctamin 2.

**Table 4:** SNPs nominally to significantly associated with diabetes type 2 and cognitive domains in the Sydney Memory and Ageing Study (MAS) and replication results from the Older Australian Twins Study (OATS).

Trait	SNP	MAS				OATS			
		Effect Allele	OR/Beta (S.E.)	p-value	N	Effect Allele	OR/Beta (S.E.)	Replication p-value (OATS)	N
Diabetes Type 2	rs2010251	T	−1.668 (0.210)	0.015	925	T	0.010 (0.029)	0.730	512
Diabetes Type 2	rs758767	A	−1.668 (0.210)	0.015	925	A	0.010 (0.029)	0.730	512
Attention/Processing Speed	rs2010251	T	−0.163 (0.073)	0.026	909	T	0.072 (0.077)	0.350	503
Attention/Processing Speed	rs758767	A	−0.163 (0.073)	0.026	909	A	0.072 (0.077	0.350	503
Attention/Processing Speed	rs2873966	A	0.194 (0.056)	0.001	909	A	−0.141 (0.073)	0.054	503
Executive function	rs2873966	A	0.149 (0.061)	0.014	851	A	−0.081 (0.076)	0.280	499
Global cognition	rs2873966	A	0.189 (0.057)	0.001	921	A	−0.14 (0.070)	0.047	505

S.E. = standard error for odds ratio (OR) or beta value.
