# Supplementary material for: Genome-wide significant results identified for plasma apolipoprotein H levels in middle-aged and older adults
Source: Sci Rep. 2016 Mar 31;6:23675. doi: 10.1038/srep23675 (PMC4814826; doi:10.1038/srep23675)
Supplement: Supplementary Information [file srep23675-s1.pdf]

SUPPLEMENTARY MATERIAL

Genome-wide significant results identified for plasma apolipoprotein H levels in middle-aged and older adults

Karen A Mather, PhD[1]*; Anbupalam Thalamuthu, PhD[1]; Christopher Oldmeadow, PhD[2]; Fei Song, PhD[1]; Nicola J Armstrong, PhD[1,3]; Anne Poljak, PhD[1,4,5]; Elizabeth G Holliday, PhD[2,6]; Mark McEvoy, PhD[2]; John B Kwok, PhD[7,8]; Amelia A Assareh, PhD[1]; Simone Reppermund, PhD[1,9]; Nicole A Kochan, PhD[1,10]; Teresa Lee, PhD[1,10]; David Ames, MD[11,12]; Margaret J Wright, PhD[13]; Julian N Trollor, MD, PhD[1,9]; Peter W Schofield, PhD[14]; Henry Brodaty, MD, DSc, FRACP, FRANZCP[1,15]; Rodney J Scott, MD[2]; Peter R Schofield, PhD, DSc[7,8]; John R Attia, MD, PhD[2]; Perminder S Sachdev, MD, PhD, FRANZCP[1,10]

[1]*Centre for Healthy Brain Ageing, School of Psychiatry, University of New South Wales, Sydney, Australia*
[2]*Hunter Medical Research Institute, University of Newcastle, Newcastle, Australia*
[3]*Mathematics and Statistics, Murdoch University, Perth, Australia*
[4]*Bioanalytical Mass Spectrometry Facility, University of New South Wales, Sydney, Australia*
[5]*School of Medical Sciences, University of New South Wales, Sydney, Australia*
[6]*Centre for Clinical Epidemiology & Biostatistics, University of Newcastle, Newcastle, Australia*
[7]*Neuroscience Research Australia, Randwick, Australia*
[8]*School of Medical Sciences, University of New South Wales, Sydney, Australia*
[9]*Department of Developmental Disability Neuropsychiatry, University of New South Wales, Sydney, Australia*
[10]*Neuropsychiatric Institute, Prince of Wales Hospital, Randwick, Australia*
[11]*National Ageing Research Institute, Melbourne, Australia*
[12]*Academic Unit for Psychiatry of Old Age, University of Melbourne, Melbourne, Australia*
[13] *Queensland Brain Institute, University of Queensland, Brisbane, Australia*
[14]*School of Medicine & Public Health, University of Newcastle, Newcastle, Australia*
[15]*Dementia Collaborative Research Centre – Assessment and Better Care, University of New South Wales, Sydney, Australia*

Supplementary Table S1. Genome-wide significant plasma ApoH GWAS results for the discovery meta-analysis and the replication sample for Model 1 (age, sex, batch assay)

SNP (rs)	Chr	BP	Effect Allele (minor)	Beta (S.E.)	*Discovery* *P*-value	Direction	Replication P-value (HCS)	Direction (HCS)	Gene	Feature
rs7211380	17	64206768	G	-1.21 (0.18)	5.67E-11	--	0.00055	-	*APOH*	3'
rs11651658	17	64198640	C	-1.19 (0.18)	8.04E-11	--	0.00092	-	NA	Near *APOH*
rs1014399	17	64196331	A	-1.16 (0.18)	1.89E-10	--	0.00153	-	NA	Near *APOH*
rs8178851	17	64215239	C	-1.13 (0.19)	2.22E-09	-	0.001216	-	*APOH*	intron
rs8178853	17	64215058	A	-1.14 (0.19)	2.26E-09	--	0.001212	-	*APOH*	intron
rs8178847	17	64216815	T	-1.13 (0.19)	2.28E-09	--	0.001233	-	*APOH*	missense (Arg-->His)
rs8178842	17	64218640	T	-1.13 (0.19)	2.39E-09	--	0.001233		*APOH*	intron
rs8178841	17	64219197	T	-1.13 (0.19)	2.41E-09	--	0.001233		*APOH*	intron
rs8178838	17	64219541	C	-1.13 (0.19)	2.45E-09	--	0.001221	-	*APOH*	intron
rs16958979	17	64223859	T	-1.13 (0.19)	2.49E-09	--	0.001208	-	*APOH*	intron
rs7213041	17	64224616	T	-1.13 (0.19)	2.51E-09	--	0.001210	-	*APOH*	intron
rs8178822	17	64225529	T	-1.13 (0.19)	2.59E-09	--	0.001237	-	*APOH*	5 UTR

Note: SNP annotation information from SNPnexus[1]

Supplementary Table S2. Suggestive plasma ApoH GWAS results for the discovery meta-analysis and the replication sample for Model 1 (age, sex, assay batch effects)

SNP (rs)	Chr	BP (snpnexus)	Effect Allele	Beta (S.E.)	*Discovery* P-value	Direction	Replication *P*-value (HCS)	Direction HCS	Gene	Feature
rs2873966	17	64211973	A	0.5441 (0.10)	8.64E-08	++	0.077199	+	*APOH*	intronic
rs11655503	17	64027346	A	-0.9518 (0.19)	4.73E-07	--	0.009783*	-	*CEP112*	intronic
rs2010251	17	64203725	T	-0.6224 (0.12)	6.04E-07	--	0.028650*	-	N/A	N/A
rs758767	17	64204591	A	-0.6214 (0.12)	6.30E-07	--	0.028656*	-	N/A	N/A
rs1420791	17	63914750	G	-0.9124 (0.19)	1.03E-06	--	0.010614*	-	*CEP112*	intronic
rs7214750	17	63903119	T	-0.8943 (0.19)	1.44E-06	--	0.015430*	-	*CEP112*	intronic
rs8064837	17	64242703	G	-0.4378 (0.09)	1.53E-06	--	0.152798	-	N/A	N/A
rs6431248	2	235202974	A	0.4521 (0.10)	4.06E-06	++	0.748016	+	N/A	N/A
rs9805211	13	30079875	C	0.6755 (0.15)	4.17E-06	++	0.555182	-	MTUS2	3’ UTR
rs12429309	13	28879332	C	0.4864 (0.11)	9.00E-06	++	0.736465	-	FLT1	intron
rs181247	17	56207731	A	0.5528 (0.12)	9.33E-06	++	0.019528*	+	N/A	N/A
rs218986	1	37168167	T	-0.4975 (0.11)	9.47E-06	--	0.630326	-	N/A	N/A
rs218984	1	37170745	G	-0.4975 (0.11)	9.47E-06	--	0.631397	+	N/A	N/A

Note: SNP annotation information from SNPnexus[1] *p<0.05

Supplementary Table S3. Results from the ApoH GWAS conditional and joint analysis (COJO) based on the meta-analysis for Model 2

					Single SNP Meta-Analysis (discovery)						Joint Analysis, LD from Sydney MAS Cohort					
**SNP**	**Chr**	**bp**	**Gene**	**Location**	**REF. ALLELE**	**FREQ**	**B**	**S.E.**	**P-value**	**VarExp**	**EST. N**	**FREQ**	**B**	**S.E.**	**P-value**	**LD_r**
rs17759236	17	51534381	N/A	N/A	G	0.404	-0.180	0.094	0.05478	0.003	991	0.404	-1.134	0.144	2.76E-15	-0.754
rs768794	17	51548399	N/A	N/A	A	0.482	-0.150	0.092	0.10150	0.002	999	0.482	-0.767	0.139	3.75E-08	0.019
rs9892443	17	61144125	*CEP112*	intronic	C	0.170	-0.062	0.127	0.62450	0.000	918	0.170	-1.471	0.161	7.96E-20	-0.223
rs8077164	17	61195107	*CEP112*	intronic	T	0.340	-0.259	0.096	0.00732	0.007	997	0.339	-1.611	0.171	5.19E-21	-0.552
rs1833348	17	61205081	*CEP112*	intronic	G	0.436	-0.156	0.097	0.10610	0.003	908	0.436	-1.330	0.164	4.57E-16	-0.586
rs746628	17	61281009	*CEP112*	intronic	C	0.300	0.180	0.104	0.08318	0.003	917	0.300	1.802	0.203	7.21E-19	0.443
rs1420802	17	61319214	*CEP112*	intronic	T	0.214	-0.195	0.113	0.08517	0.003	970	0.214	-1.247	0.154	7.04E-16	0.016
rs7214750	17	61333581	*CEP112*	intronic	T	0.063	-0.904	0.187	0.00000	0.021	989	0.063	-14.352	0.492	2.7E-187	-0.199
rs997652	17	61344827	*CEP112*	intronic	T	0.403	-0.182	0.093	0.05089	0.004	995	0.403	-2.561	0.196	4.73E-39	-0.211
rs9893012	17	61375061	*CEP112*	intronic	G	0.072	0.613	0.177	0.00053	0.011	985	0.072	13.621	0.498	1.5E-164	-0.212
rs1838104	17	61414011	*CEP112*	intronic	C	0.433	-0.201	0.092	0.02934	0.004	1000	0.433	-4.817	0.269	7.1E-72	-0.258
rs8080448	17	61470360	*CEP112*	intronic	A	0.092	-0.638	0.169	0.00016	0.015	858	0.092	-15.289	0.483	2.4E-220	-0.308
rs12602270	17	61531072	*CEP112*	intronic	T	0.475	0.213	0.091	0.01894	0.005	1015	0.475	5.871	0.243	2.8E-129	-0.345
rs9897921	17	61555230	*CEP112*	intronic	C	0.112	0.597	0.155	0.00012	0.016	857	0.112	13.844	0.437	1.1E-220	-0.285
rs9904627	17	61555793	*CEP112*	intronic	T	0.430	0.156	0.093	0.09259	0.003	993	0.430	2.553	0.215	2.08E-32	-0.255
rs12949444	17	61564331	*CEP112*	intronic	A	0.235	0.427	0.115	0.00021	0.014	860	0.235	4.768	0.230	1.26E-95	0.422
rs4791083	17	61600257	*CEP112*	intronic	C	0.445	0.001	0.100	0.99420	0.000	847	0.445	-2.341	0.165	1.03E-45	-0.334
rs1014398	17	61626723	N/A	N/A	C	0.105	-0.428	0.151	0.00448	0.008	976	0.105	-13.796	0.384	6.8E-283	0.400
rs2010251	17	61634187	N/A	N/A	T	0.162	-0.610	0.126	1.26E-06	0.022	950	0.162	-2.607	0.218	6.7E-33	0.586
rs7211380	17	61637230	*APOH*	3' downstream	G	0.062	1.205	0.185	8.15E-11	0.037	1011	0.062	21.124	0.483	<1.1E-220	-0.196
rs4791078	17	61640476	*APOH*	intronic	A	0.401	-0.272	0.100	0.00619	0.008	873	0.401	-3.565	0.231	1.39E-53	-0.466
rs7212060	17	61645860	*APOH*	intronic	T	0.298	-0.295	0.103	0.00403	0.008	941	0.298	-5.443	0.242	4.3E-112	-0.355
rs9892748	17	61667403	N/A	N/A	C	0.240	-0.414	0.108	0.00012	0.014	973	0.240	-8.258	0.263	4.1E-217	-0.209
rs735866	17	61670208	N/A	N/A	G	0.114	-0.016	0.138	0.90700	0.000	1091	0.114	2.334	0.298	4.88E-15	-0.162
rs17706845	17	61740647	*PRKCA*	intronic	G	0.418	0.263	0.094	0.00542	0.007	958	0.418	2.983	0.216	2.74E-43	-0.233
rs4527055	17	61760907	*PRKCA*	intronic	C	0.090	0.016	0.158	0.92010	0.000	1024	0.090	2.725	0.328	9.13E-17	-0.237
rs9903921	17	61762063	*PRKCA*	intronic	T	0.354	0.345	0.098	0.00041	0.012	949	0.354	4.308	0.208	6.21E-95	0

Notes: COJO analysis implemented in the GCTA program [2]; Joint analysis used the Sydney MAS cohort as the reference sample for LD estimation.

Supplementary Table S4. List of the ten SNPs that were either genome-wide significant or suggestive in the prior Athanasiadis et al. ApoH GWAS [3]

**SNP**	**Chr**	**P-value**	**SNP type**	**Closest gene**
rs836132	11	4.57×10^{-8}	Intergenic	*LEF5*
rs963167	11	1.22×10^{-7}	Intronic	*SCUBE2*
rs836135	11	1.39×10^{-7}	Intergenic	*ELF5*
rs2647528	11	3.30×10^{-7}	Intronic	*SCUBE2*
rs7209395	17	7.20×10^{-7}	Intronic	*CEP112*
rs10048158	17	1.12×10^{-6}	Intergenic	*APOH*
rs2319125	17	1.37×10^{-6}	Intronic	*CEP112*
rs4925295	20	1.63×10^{-6}	Intronic	*CDH4*
rs193741	5	1.88×10^{-6}	Intergenic	*CDH10/CDH9*
rs11190179	10	1.91×10^{-6}	Intergenic	*SLC25A28*

Note: Adapted from Athanasiadis et al. Table 1[3].

Supplementary References

1. Dayem Ullah, A. Z., Lemoine, N. R. & Chelala, C. A practical guide for the functional annotation of genetic variations using SNPnexus. *Brief Bioinform*. **14**, 437-447 (2013).
2. Yang, J. *et al.* Conditional and joint multiple-SNP analysis of GWAS summary statistics identifies additional variants influencing complex traits. *Nat Genet*. **44**, 369-375, S361-363 (2012).
3. Athanasiadis, G. *et al.* Genetic determinants of plasma beta(2)-glycoprotein I levels: a genome-wide association study in extended pedigrees from Spain. *J Thromb Haemost*. **11**, 521-528 (2013).
